# *ROS1-GOPC/FIG*: a novel gene fusion in hepatic angiosarcoma

**DOI:** 10.18632/oncotarget.26521

**Published:** 2019-01-04

**Authors:** Eric I. Marks, Sahithi Pamarthy, Don Dizon, Ari Birnbaum, Evgeny Yakirevich, Howard Safran, Benedito A. Carneiro

**Affiliations:** ^1^ Division of Hematology-Oncology, Lifespan Cancer Institute, Warren-Alpert Medical School of Brown University, Providence, RI, USA; ^2^ Atrin Pharmaceuticals, Pennsylvania Biotechnology Center, Doylestown, PA, USA; ^3^ Department of Pathology, Lifespan Cancer Institute, Warren-Alpert Medical School of Brown University, Providence, RI, USA

**Keywords:** ROS1, GOPC, FIG, angiosarcoma, Targeted Therapy

## Abstract

Hepatic angiosarcoma (HAS) is a rare and highly lethal malignancy with few effective systemic treatments. Relatively little is known about the genetic abnormalities that drive this disease. As a result, there has been minimal progress towards applying targeted therapies to the treatment of HAS. We describe the first reported case of a patient with HAS that harbored a fusion of *ROS1* with *GOPC/FIG*. Similar to other rearrangements involving *ROS1*, the resulting fusion protein is believed to act as a major driver of carcinogenesis and may be subject to inhibition by drugs that target *ROS1* such as crizotinib. We then queried the MSK-IMPACT clinical sequencing cohort and cBioportal datasets, demonstrating the previously unknown prevalence of *ROS1-GOPC* fusions in soft tissue sarcomas and hepatobiliary cancers. Amplification of these genes was also found to correlate with reduced overall survival. This is followed by a review of the role played by *ROS1* rearrangements in cancer, as well as the evidence supporting the use of targeted therapies against the resulting fusion protein. We suggest that testing for ROS1 fusion and, if positive, treatment with a targeted therapy could be considered at the time of diagnosis for patients with angiosarcoma. This report also highlights the need for further investigation into the molecular pathophysiology of this deadly disease.

## INTRODUCTION

Hepatic angiosarcoma (HAS) is a rare disease, with an annual incidence of approximately 200 cases worldwide [1]. It accounts for 2% of all primary malignancies of the liver and is the third most common type of liver cancer [2]. While exposure to vinyl chloride, arsenic, thorotrast, and radium have been identified as risk factors for the development of HAS, most cases arise in patients without exposure [1, 2]. Advanced HAS carries a poor prognosis, with 2 year overall survival (OS) of approximately 33%. This is related to presentation at advanced stages of disease, high rate of spontaneous tumor rupture resulting in intra-abdominal hemorrhage, and limited response to chemotherapy or radiation [2]. The standard treatment for patients with localized disease is complete surgical resection, often followed by adjuvant chemotherapy. While there is no universally accepted standard treatment in the metastatic setting, commonly used chemotherapies include doxorubicin, paclitaxel, or gemcitabine [3].

Although targeted therapies have revolutionized the treatment of other malignancies, such as non-small cell lung cancer (NSCLC) or breast cancer, there has been little progress towards applying these treatments to angiosarcoma. This is partially due to the limited knowledge regarding the landscape of genomic abnormalities from small cohorts of specimens that examined a select panel of genes. Frequent alterations of the RAS-RAF-MAPK pathway, *TP53* and *CDKN2A*/*p16* genes have been observed [4]. Increased expression of vascular endothelial growth factor (VEGF) and its receptor (VEGFR) is also common and supported clinical investigation of drugs that inhibit angiogenesis such as bevacizumab (a monoclonal antibody against VEGF-A) [5], sorafenib (tyrosine kinase inhibitor (TKI) that targets VEGFR and BRAF) [6, 7] and pazopanib (TKI that targets VEGFR, fibroblast growth factor receptor, and platelet derived growth factor) [8]. Tumor responses to these agents are seen in 10–25% of patients and progression-free survival (PFS) limited to 1–4 months.

Abnormalities in the RAS-RAF-MAPK pathway have been targeted with the mTOR (mammalian target of rapamycin) inhibitor everolimus. Two patients with angiosarcoma were treated with everolimus and both had a partial response (PR) lasting 6–12 months [9]. In a subgroup analysis of 3 patients with angiosarcoma enrolled in a multicenter phase II trial of everolimus for metastatic soft tissue sarcomas, one had PR and another stable disease (SD) [10]. Both of these patients had continued disease control for at least 16 weeks.

We describe the first case of HAS harboring a fusion of *ROS1* with *GOPC/FIG* and review of the role of *ROS1* rearrangements in cancer and evidence supporting the use of therapeutics that target *ROS1*.

## CLINICAL CASE

**Figure 1:**
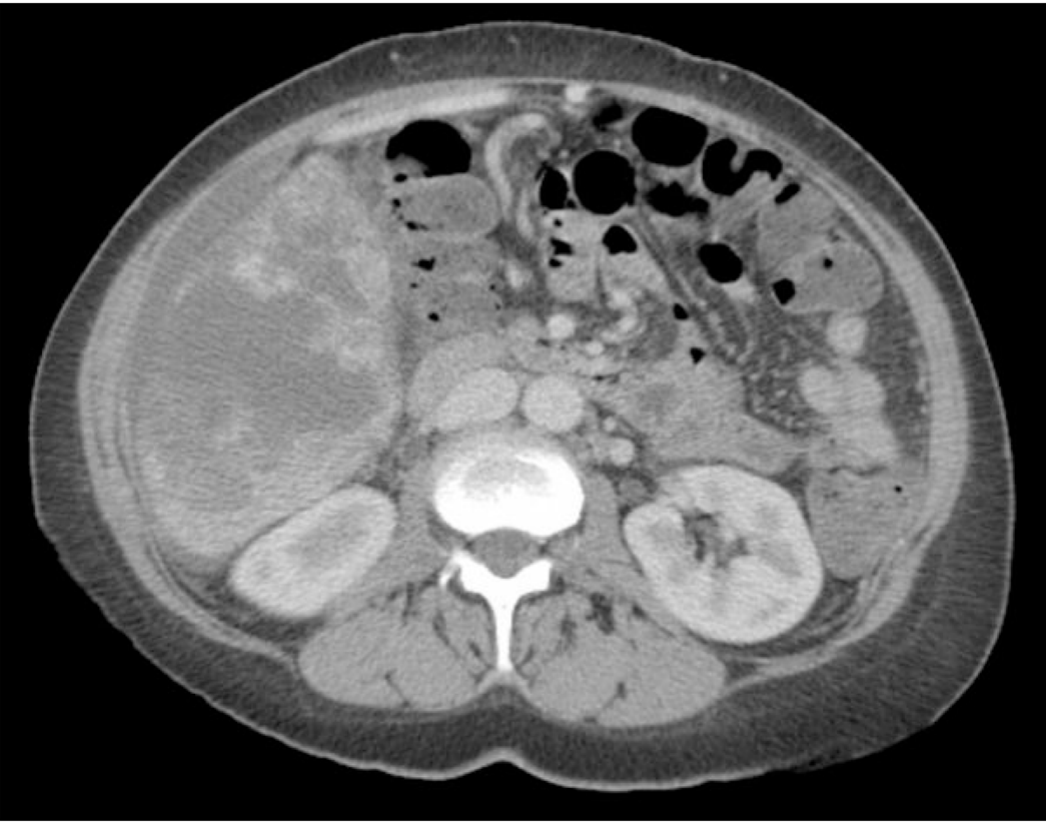
CT scan of the abdomen and pelvis with intravenous contrast showing multiple masses throughout the liver, later biopsy-proven hepatic angiosarcoma

A 50 year-old woman presented to her primary care physician for evaluation of a 2 week history of right upper quadrant abdominal pain and weight loss. CT of the abdomen and pelvis revealed multifocal hepatic disease and a dominant 7 cm lesion in the right lobe of the liver (Figure 1). CT scans of chest and brain showed no evidence of extrahepatic disease.

**Figure 2:**
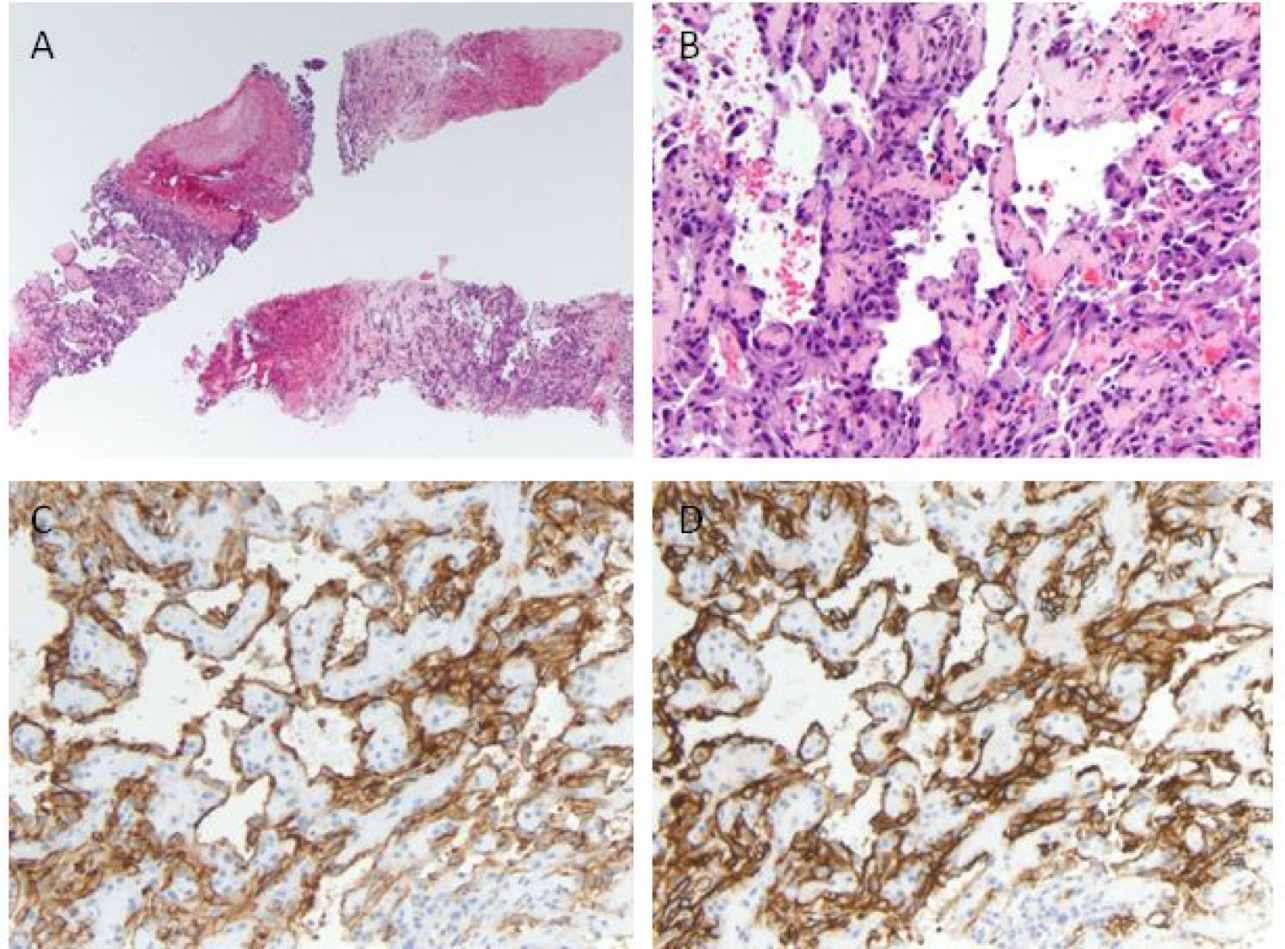
Histologic and immunohistochemical analysis of the liver tumor (**A**) Low-power view of the biopsy cores containing hemorrhagic and necrotic tumor, hematoxylin and eosin (H&E), original magnification ×40. (**B**) On higher magnification tumor cells form irregular anastomosing vascular channels lined by cells with hyperchromatic atypical nuclei (H&E, original magnification ×200). Tumor cells express the endothelial markers CD31 (**C**), and CD34 (**D**).

Percutaneous liver biopsy showed an extensively hemorrhagic and necrotic tumor composed of irregular, anastomosing vascular channels lined with atypical cuboidal to flattened endothelial cells with irregular hyperchromatic nuclei (Figure 2). Occasional mitoses were identified in vascular lining. The neoplastic cells were positive for vascular endothelial markers CD31 and CD34, but negative for cytokeratin E1/AE3, cytokeratins 7 and 20, hep-par1, AFP, and CA19.9. Proliferation index as detected by Ki-67 immunostaining was variable, ranging from <10% to focal areas of 40%. These morphologic features and immunophenotype were consistent with diagnosis of HAS.

Before starting therapy, patient had an acute decrease in hemoglobin (Hgb) from 8 to 6.5 and repeated CT scan that showed progression of dominant lesion to 12 cm, ascites and a small area of subcapsular hemorrhage compared to scan one month prior. The patient underwent hepatic artery embolization with post-procedural stabilization of Hgb and received chemotherapy with paclitaxel. Shortly thereafter, the patient experienced gradual deterioration of performance status with progressive abdominal pain, ascites and lower extremity edema. She chose to not receive additional cancer-directed therapy and pursued hospice care. The patient expired 2 months after initial diagnosis.

Comprehensive genomic profiling (CGP) performed on liver biopsy specimen revealed a *ROS1* rearrangement involving *GOPC* that had not been previously described in HAS. [11]. CGP of the liver biopsy specimen was performed in a Clinical Laboratory Improvement Amendments (CLIA)-certified pathology laboratory (Foundation Medicine, Cambridge, MA), as previously described [11, 12]. In brief, ≥50 ng DNA was extracted from 40 microns of tumor sample in formalin-fixed, paraffin-embedded tissue blocks. Next generation sequencing was performed and targeted all coding exons in 405 cancer-related genes plus select introns within 31 genes that often display rearrangements in malignancy (FoundationOne) using Illumina HiSeq (Illumina, San Diego, CA) technology [13].

Additional abnormalities included mutations in *MLL2* and *PRDM1* genes, *CDKN2A* loss, and a low overall tumor mutational burden (4 mutations per megabase). Variants of unknown significance were noted in the following genes *BRSK1, JARID2, KIT, MLH1, PPP2R1A,* and *ZNF217.* Since these results became available only after the patient's decline and subsequent transition to hospice care, she was unable to receive targeted therapy against *ROS1*.

## LANDSCAPE OF *ROS1* AND *GOPC* GENOMIC ALTERATIONS IN CANCERS

**Figure 3:**
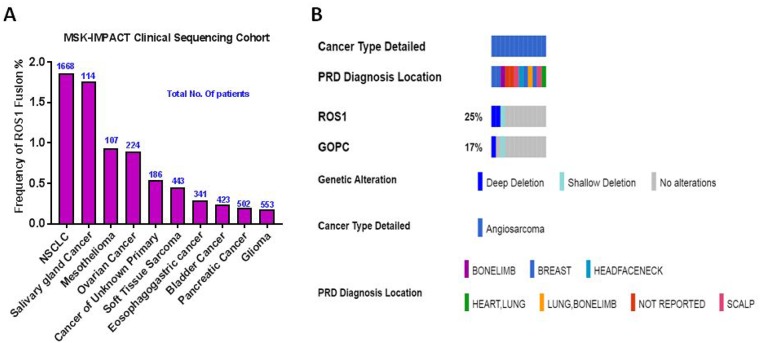
Frequency of *ROS1* gene fusions in cancer (**A**) Combined *ROS1* fusion frequency with all known gene partners in a large pan-cancer dataset. Number of patients for each cancer subtype is shown in blue. NSCLC: Non-Small Cell Lung Cancer. (**B**) Oncoprint of *ROS1* and *GOPC* copy number alterations in a small angiosarcoma (*n* = 12) project dataset taken from cBioportal is shown. PRD Diagnosis refers to patient reported response to initial diagnosis location of angiosarcoma.

To investigate the frequency of *ROS1* gene fusions among multiple cancer types, we queried MSK-IMPACT Clinical Sequencing Cohort [14], a pan cancer cohort of tumor samples with sequencing and copy number alterations (CNA) data (10336 patients/10945 samples) available at cBioportal cancer genomics database (www.cbioportal.org). *ROS1* gene fusions with any gene partner occurred among 40 patients (0.4%) with the highest frequency of occurrences in non-small cell lung cancer (NSCLC; 31 of 1668 cases:1.8%) and salivary gland cancer (2 of 114 cases: 1.7%). Among the two cases of soft tissue sarcoma identified, one patient with perivascular epithelioid cell tumor had both *ROS1-NETO1* and *SLC4A1-ROS1* fusions while the other patient with synovial sarcoma carried *COL4A3BP-ROS1* fusion. In this analysis we found only two cases carrying *ROS1-GOPC* fusions: high grade glioma and cancer of unknown primary (Figure 3A). The list of gene fusion partners with *ROS1* from this dataset is summarized in Table 1. Specifically, a small cohort of angiosarcoma project dataset in cBioportal showed homodeletion of *ROS1* in two out of 12 sequenced patients. One of the cases was a breast angiosarcoma patient with co-occurring *GOPC* deletion at 6q22.1 (Figure 3B).

**Table 1:** ROS1 gene fusion partners in various cancer subtypes

Gene fusion pair	Cancer type	Total No. of patients
CD74-ROS1	Lung Adenocarcinoma	16
EZR-ROS1	Lung Adenocarcinoma	6
ROS1-SDC4	Lung Adenocarcinoma	4
GOPC-ROS1	High-Grade Glioma, NOS	2
	Cancer of Unknown Primary	
SLC34A2-ROS1	Lung Adenocarcinoma	2
SLC4A10-ROS1	Perivascular Epithelioid Cell Tumor	1
SLC4A4-ROS1	Pancreatic Adenocarcinoma	1
SLC6A17-ROS1	Lung Adenocarcinoma	1
ROS1-NETO1	Perivascular Epithelioid Cell Tumor	1
ROS1-SLC16A10	Bladder Urothelial Carcinoma	1
TMEM181-ROS1	Pleural Mesothelioma, Epithelioid Type	1
TPM3-ROS1	Lung Adenocarcinoma	1
C6orf204-ROS1	Salivary Carcinoma	1
COL4A3BP-ROS1	Synovial Sarcoma	1
GOLGB1-ROS1	Serous Borderline Ovarian Tumor	1
ROS1-FAM135B	Lung Adenocarcinoma	1
ROS1-HS3ST5	Stomach Adenocarcinoma	1

List of ROS1 fusion partners and total number of patients identified with each gene fusion pair is shown. Data is extracted from MSK-IMPACT Clinical Sequencing Cohort at cBioportal. Gene abbreviations in order of appearance in table – CD74, Cluster of Differentiation 74; EZR, Ezrin; ROS1, ROS Proto-Oncogene 1; SDC4, Syndecan-4; GOPC, Golgi Associated PDZ And Coiled-Coil Motif Containing; SLC34A2, Solute Carrier Family 34 Member 2; SLC4A10, Solute Carrier Family 4 Member 10; SLC4A4, Solute Carrier Family 4 Member 4; SLC6A17, Solute carrier family 6 member 17; NETO1, Neuropilin And Tolloid Like 1; SLC16A10, Solute Carrier Family 16 Member 10; TMEM181, Transmembrane Protein 181; TPM3, Tropomyosin 3; C6orf204, chromosome 6 open reading frame 204; COL4A3BP, Collagen type IV alpha-3-binding protein; GOLGB1, Golgin B1; FAM135B, Family With Sequence Similarity 135 Member B; HS3ST5, Heparan Sulfate-Glucosamine 3-Sulfotransferase 5.

**Figure 4:**
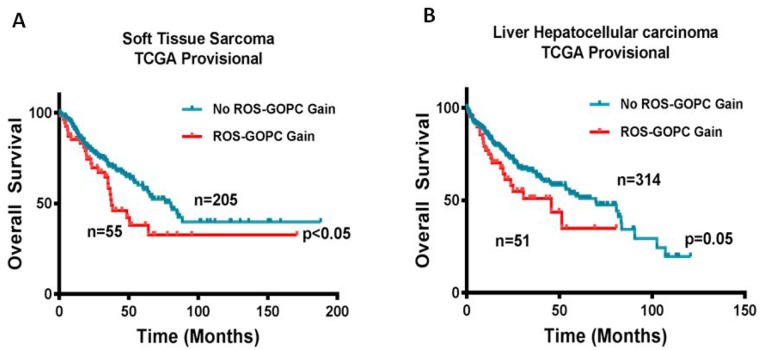
Gain in ROS1 and GOPC copy number is associated with poor overall survival Kaplan–Meier survival curves for overall survival of patients with or without co-occurring ROS1 and GOPC copy number gain from (**A**) TCGA Soft Tissue Sarcoma dataset and (**B**) TCGA Hepatocellular carcinoma provisional dataset from cBioportal database were generated. Total number of patients in the two categories is shown. ^*^P ≤ 0.05 for Log-rank (Mantel Cox) test.

To further explore the incidence of *ROS1* and *GOPC* concurrent alterations in primary hepatobiliary tumors and soft tissue sarcomas, we queried available datasets in cBioportal. In TCGA sarcoma dataset (265 patients with soft tissue sarcomas and 9 patients with nerve sheath tumors), we identified concurrent gene amplification or gain of *ROS1* and *GOPC* involving 6q22.1 in 53 (21%) of the patients. In the TCGA hepatocellular carcinoma dataset, we identified 51 (14%) of 366 sequenced patients, of which 50 had low level gain and 1 patient had high level amplification of both genes. Among the 51 patients with concurrent gain of *ROS1* and *GOPC*, we identified a patient with hepatocellular carcinoma plus intrahepatic cholangiosarcoma. Notably, Kaplan–Meier survival estimate showed a poor overall survival in both soft tissue sarcoma and hepatocellular carcinoma patients with *ROS1* and *GOPC* gain/amplification (Figure 4). This database analysis highlights the previously unknown involvement of *ROS1* and *GOPC* copy number alterations in soft tissue sarcomas and hepatic cancers.

## DISCUSSION

*ROS1* is a proto-oncogene located on the long arm of chromosome 6 and encodes a receptor tyrosine kinase involved in the regulation of cancer cell growth and differentiation. *ROS1* is often involved in genomic rearrangements resulting in constitutionally active kinases that stimulate multiple pathways such as JAK-STAT, PI3K-AKT-mTOR, and RAS-RAF-MEK-ERK [15]. Fusion products of *ROS1* have been observed in a variety of types of cancer, including tumors of the lung, gastrointestinal tract, hepatobiliary tree, and central nervous system [15]. Fusion partners that participate in ROS1 rearrangements include *CD74*, *SLC34A2*, *SDC4* in addition to *GOPC/FIG*, which was observed in this patient's tumor [15]. *ROS1* rearrangement with another gene (*CEP85L*) has been previously described in angiosarcoma (primary site unknown) [16].

The *GOPC/FIG* (Fused In Glioblastoma) gene also resides on the short arm of chromosome 6 and encodes a protein displaying coiled-coil and PDZ domains. Through interaction with the PDZ domain (structural domain that recognize amino acid motifs), this protein localizes to the Golgi apparatus where it facilitates the intracellular trafficking of proteins to the cell surface and lysosomes [17, 18]. Among the proteins under control of GOPC/FIG are the cystic fibrosis transmembrane conductance regulator (CFTR), *frizzled* 5 and 8, beta-1-adrenergic receptor, cadherin 23, and somatostatin receptor subtype 5 [18].

*GOPC/FIG* was initially named following its discovery as a *ROS1* fusion partner in a patient-derived astrocytoma cell line [17]. As a result of a 240 kb deletion on 6q21, exon 36 of *ROS1* was fused to exon 3 of *GOPC* [17, 19]. Since this initial “long” fusion was described, a second “short” isoform that attaches exon 7 of *FIG* to exon 35 of *ROS1* has also been identified [19]. *ROS1-GOPC* fusions have been observed in anaplastic astrocytoma [20], NSCLC [21], ovarian serous tumor [22], cholangiocarcinoma [19], and acral lentiginous melanoma [23]. Similar to other *ROS1* fusions, *ROS1- GOPC* fusion proteins are believed to represent major drivers of carcinogenesis [19, 24]. Preclinical research has demonstrated that these gene fusions induce tumorigenesis both *in vitro* and *in vivo* [19, 24].

The identification of a *ROS1- GOPC* fusion has significant clinical implications due to the established anti-cancer activity of TKIs in tumors that carry *ROS1* rearrangements. Crizotinib is a small molecule inhibitor of multiple tyrosine kinases, most notably *ALK* and *ROS1*. In a study of 50 patients with NSCLC and *ROS1* rearrangement, treatment with crizotinib resulted in a 72% response rate and median PFS of 19.2 months [25]. Crizotinib has since gained FDA approval in NSCLC with *ROS1* rearrangement. Although the prevalence of *ROS1-GOPC* fusion is too low for a dedicated clinical investigation, research in NSCLC cell lines has demonstrated *in-vitro* inhibition of cell growth following exposure to crizotinib [21].

While most of the research supporting *ROS1* inhibition was conducted in NSCLC, there is anecdotal evidence of efficacy in other diseases. One patient with heavily pretreated acral lentiginous melanoma carrying a *ROS1-GOPC* fusion was treated with entrectinib (TKI with activity against tropomyosin receptor kinase (*Trk*), *ROS1* and *ALK*), leading to a PR with 38% reduction in tumor burden at 3 months and 55% at 11 months [23]. The response was ongoing at the time of publication.

Unfortunately, the presence of a *ROS1* fusion in this patient's tumor was identified only after she had experienced significant decline in her performance status and quality of life. As a result, she did not receive targeted therapy against *ROS1*. Such treatment has a rather modest side effect profile and has produced prolonged responses in patients with other malignancies. This case illustrates the importance of obtaining comprehensive genetic and molecular profiling early in the course of disease. This is particularly crucial for malignancies that are often resistant to standard treatments such as angiosarcoma, or frequently harbor actionable mutations such as *ROS1* fusions. Although this is the first report of *ROS1-GOPC* fusion in HAS, database analysis highlights the previously unknown frequency of *ROS1* and *GOPC* molecular alterations in soft tissue sarcomas and hepatic cancers. This includes both fusions, which typically confer sensitivity to treatment with TKI’s, and copy number alterations of *ROS1*. Further study is needed to better characterize the relationship between amplification of *ROS1* and response to targeted inhibitors. In addition, our analysis found amplification of these genes to correlate with reduced overall survival. Research in NSCLC has been inconclusive on this subject, with one study finding *ROS1* copy number gain (CNG) to correlate with impaired disease-free and overall survival [26] but another finding no significant relationship [27]. However, when considered in tandem with the findings that *ROS1* CNG does not always correlate to over-expression of the protein [26] and the most common mechanism of *ROS1* CNG is polysomy of chromosome 6 [27], it seems most likely that *ROS1* CNG is a surrogate marker of an aggressive tumor with complex karyotype.
