# Addendum: *ROS1-GOPC/FIG*: a novel gene fusion in hepatic angiosarcoma

**DOI:** 10.18632/oncotarget.28448

**Published:** 2023-05-26

**Authors:** Eric I. Marks, Sahithi Pamarthy, Don Dizon, Ari Birnbaum, Evgeny Yakirevich, Howard Safran, Benedito A. Carneiro

**Affiliations:** ^1^Division of Hematology-Oncology, Lifespan Cancer Institute, Warren-Alpert Medical School of Brown University, Providence, RI, USA; ^2^Atrin Pharmaceuticals, Pennsylvania Biotechnology Center, Doylestown, PA, USA; ^3^Department of Pathology, Lifespan Cancer Institute, Warren-Alpert Medical School of Brown University, Providence, RI, USA; ^*^These authors contributed equally to this work

**This article has an addendum:** Informed patient consent to an IRB-approved research protocol was obtained.

Original article: Oncotarget. 2019; 10:245–251. 245-251. https://doi.org/10.18632/oncotarget.26521

